# Supplementary material for: Safety and Immunogenicity of Recombinant Bacille Calmette-Guérin Strain VPM1002 and Its Derivatives in a Goat Model
Source: Int J Mol Sci. 2023 Mar 14;24(6):5509. doi: 10.3390/ijms24065509 (PMC10058566; doi:10.3390/ijms24065509)
Supplement: Supplementary file 1 [file ijms-24-05509-s001.zip › ijms-2210276-Supplementary.pdf]

Supplementary data

Table S1: Bacterial culture of nasal swabs of vaccinated or mock-treated goats.

sample	group	goat no.	sampling day (dpv) -14	0	28	56	84	112
nasal swab	mock-treated	0008	-	-	-	-	-	-
		0031	-	-	-	-	-	-
		0014	-	-	-	-	-	-
		0023	-	-	-	cont	-	-
		0029	-	-	-	-	-	-
		0030	-	-	-	-	-	-
	BCG	0007	-	-	-	-	-	-
		0013	cont	-	-	-	-	-
		0015	-	-	-	-	-	-
		0022	-	-	-	-	-	-
		0027	-	-	-	-	-	-
		0034	-	-	-	-	-	cont
	VPM1002	0001	-	-	-	-	-	-
		0002	-	-	-	-	-	-
		0009	-	-	-	-	-	-
		0016	-	-	-	-	-	cont
		0017	-	-	-	-	-	-
		0024	-	-	-	-	-	-
	NUOG	0005	-	-	-	-	-	-
		0006	-	-	-	-	-	-
		0011	-	-	-	-	-	-
		0020	-	-	-	-	-	-
		0021	-	-	-	-	-	-
		0026	-	-	-	-	-	-
	PDX	0003	-	-	-	-	-	-
		0004	-	-	-	-	-	-
		0010	-	-	-	-	-	-
		0018	-	-	-	-	-	-
		0019	cont	-	-	-	-	-
		0025	-	-	-	-	-	-

(- no growth, cont contaminated)

Table S2: Mycobacterial cultures of tissue samples after necropsy of vaccinated or mock-treated goats (127 days post vaccination).

Group	Goat No.	A	B	C	D	E	F	G	H	J	K	L	M	N	O	P	Q	R	S	T	U	V	W	X
mock-treated	8	-	-	-	-	-	-	-	-	-	-	-	-	-	-	-	-	-	-	-	-	-	-	-
	14	-	-	-	-	-	-	nd	-	-	-	-	-	-	-	-	-	-	#	-	-	-	nd	-
	23	-	-	-	-	nd	-	-	-	-	-	-	-	-	-	-	-	-	-	-	-	-	-	-
	29	-	-	-	-	-	-	-	-	-	-	-	-	-	-	-	-	-	-	-	-	-	nd	-
	30	-	-	-	-	-	-	-	-	-	-	-	-	-	-	-	-	-	-	-	-	-	-	nd
	31	-	§	-	-	-	nd	-	-	-	-	-	-	-	-	-	-	-	-	-	-	-	nd	-
BCG	7	+	-	-	-	nd	-	-	-	+	-	-	-	-	-	-	-	-	-	-	-	-	-	-
	13	-	-	-	-	-	-	nd	-	-	-	-	-	-	-	-	-	-	-	-	-	-	nd	-
	15	+	-	-	-	-	-	-	-	-	-	-	-	-	-	-	-	cont	-	-	-	-	#	-
	22	+	-	-	-	-	-	-	-	-	-	-	-	-	-	-	-	-	-	-	-	-	-	-
	27	-	-	-	-	-	-	-	-	-	-	-	-	-	-	-	-	-	#	-	-	-	nd	-
	34	-	-	-	-	-	-	-	-	-	-	-	-	-	-	-	-	-	-	-	-	-	-	-
VPM1002	1	-	-	-	-	nd	-	-	cont	-	-	-	-	-	-	-	-	-	-	-	-	-	-	-
	2	-	-	-	-	nd	-	-	-	-	-	-	-	-	-	-	-	-	-	-	-	-	-	-
	9	-	-	-	-	-	-	-	-	-	-	-	-	-	-	-	-	-	-	-	-	-	-	-
	16	+	-	-	-	-	-	-	nd	-	-	-	-	-	-	-	-	-	-	-	-	-	nd	-
	17	+	-	-	-	-	-	-	-	-	-	-	-	-	-	-	-	-	-	-	-	-	nd	-
	24	-	-	-	-	nd	-	-	-	-	-	-	-	-	-	-	-	-	-	-	-	-	nd	-
NUOG	5	-	-	-	-	-	-	-	-	#	-	-	-	-	-	-	-	-	-	#	-	-	-	-
	6	-	-	-	-	-	-	-	-	-	-	-	-	-	-	-	-	-	-	-	-	-	-	-
	11	-	-	-	-	-	-	-	-	-	-	-	-	-	-	-	-	-	-	-	-	-	nd	-
	20	+	-	-	-	-	-	-	-	-	-	-	-	-	-	-	-	-	-	-	-	-	nd	-
	21	-	-	-	-	-	-	nd	-	-	-	-	-	-	-	-	-	-	-	-	-	-	nd	-
	26	-	-	-	-	-	-	-	-	-	-	-	-	-	-	-	-	-	-	-	-	-	-	-
PDX	3	-	-	-	-	-	-	-	-	-	-	-	-	-	-	-	-	-	-	-	-	-	nd	-
	4	-	-	-	-	-	-	nd	-	-	-	-	-	-	-	-	-	-	-	#	-	-	nd	-
	10	-	-	-	-	-	-	-	-	-	-	-	-	-	-	-	-	-	-	-	-	-	nd	-
	18	-	-	-	-	-	-	-	-	-	-	-	-	-	-	-	-	-	-	-	-	-	-	-
	19	-	-	-	-	-	-	-	-	-	-	-	-	-	-	-	-	-	-	-	-	-	nd	-
	25	-	-	-	-	-	-	-	-	-	-	-	-	-	-	-	-	-	-	-	-	-	nd	-

A	Skin at vaccination site	N	Kidney	-	negative
B	Contralateral skin site	O	*Ln renales*	+	*M. bovis*
C	*Ln cervicalis superficialis* L	P	Heart (rV)	#	*M. gordonae*
D	*Ln cervicalis superficialis* R	Q	Tonsil	§	*M. septicum/M. peregrinum*
E	*Ln axillaris profundus* L	R	*Ln retroph medialis*	nd	not done
F	*Ln axillaris profundus* R	S	Jejunal Peyer´s Patch	cont	contaminated
G	Lung - dors. caud. lobe L	T	Ileum		
H	*Ln tracheobronchialis* L	U	*Ln mesenterialis cran.*		
J	*Lnn mediastinales*	V	*Ln ileocolici*		
K	Liver	W	Lung lesioned		
L	*Ln hepatici*	X	Bone marrow		
M	Spleen				

Table S3: Primers used for Polymerase Chain Reaction (PCR)-based genotyping of VPM1002 and its derivatives and expected PCR product..

			product size (in bp)		
**target**	**primer**	**sequence (5´-3´)**	**VPM1002**	**PDX**	**NUOG**
LLO	Hlynt.fw	AATGCTGTACCAAATTTCGCAATTAAT	500	500	500
	Hlyint.rev	CTAAGACGCCAATCGAAAAGAAACA			
Pdx	Pdx1loc.fw	ATGGATCCTGCAGGTAACCCAGC	900	300	900
	Pdx1loc.rev	ATGACCGACAGCGATCTCGTCC			
NuoG	nuoG-flank.fw	CACTGACATCCGGGTAGGCC	2400	2400	300
	nuoG-flank.rev	TCATGAGCCCGCTCCGAT			

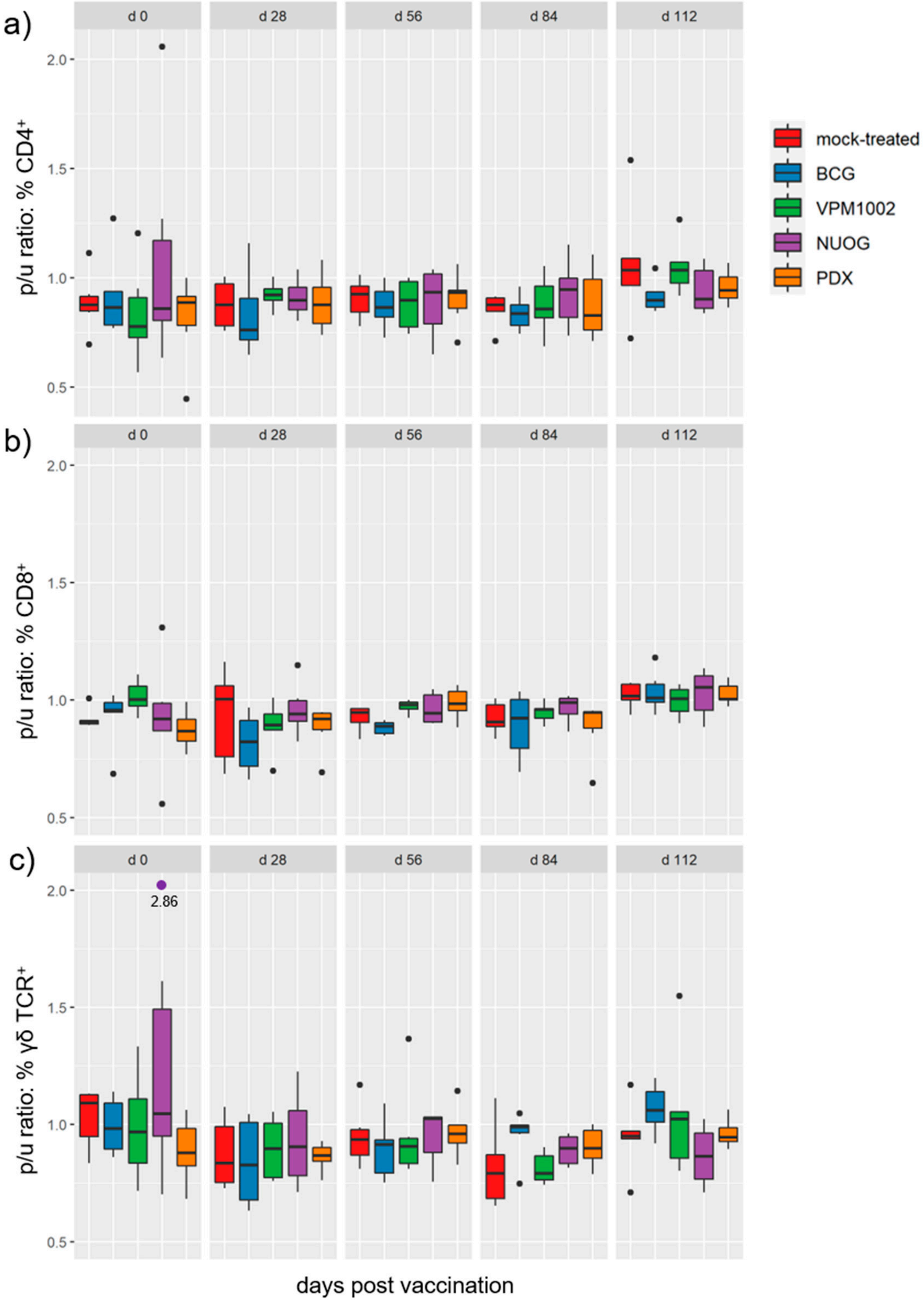

Figure S1: Percentage of (a) CD4⁺, (b) CD8⁺ and (c) γδ T cells after *in vitro* stimulation with bovine purified protein derivative (bPPD; TB antigen) of vaccinated or mock-treated goats. Box plots represent values of the p/u ratio ($\frac{\text{bPPD stimulated}}{\text{unstimulated}}$). There were no significant differences between groups (Kruskal-Wallis test).

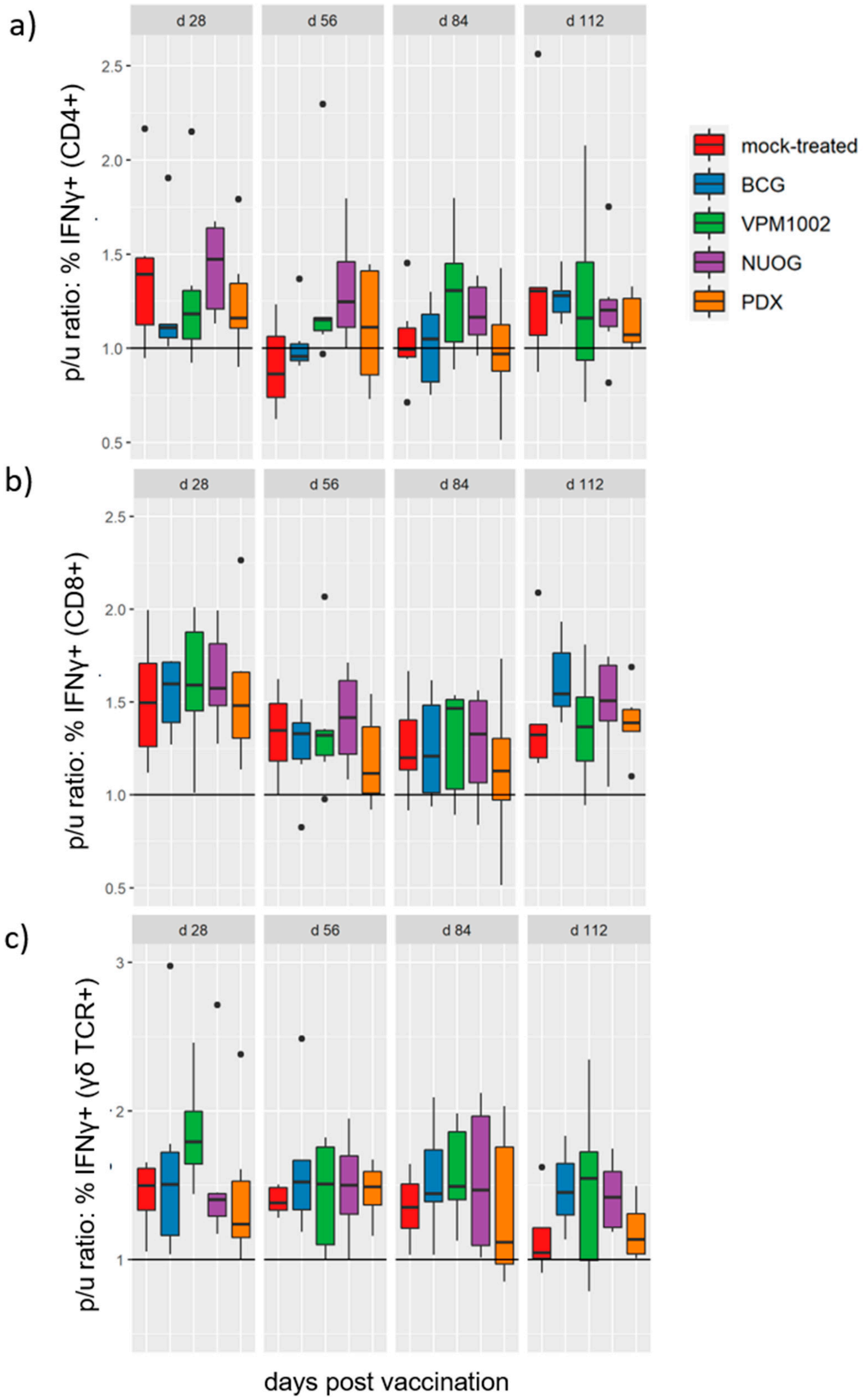

Figure S2: Percentage of cells within major T cell subsets with detectable levels of intracellular Interferon gamma after *in vitro* stimulation with bPPD of vaccinated or mock-treated goats. Box plots represent values of the p/u ratio ($\frac{\text{bPPD stimulated}}{\text{unstimulated}}$) of % IFN-$\gamma^{+}$ cells within the population of (a) $CD4^{+}$ T cells, (b) $CD8^{+}$ T cells, and (c) γδ T cells. There were no significant differences between groups (Kruskal-Wallis test).
